# Tumor Vessel Development and Expansion in Ewing's Sarcoma: A Review of the Vasculogenesis Process and Clinical Trials with Vascular-Targeting Agents

**DOI:** 10.1155/2011/165837

**Published:** 2011-06-16

**Authors:** Keri S. Stewart, Eugenie S. Kleinerman

**Affiliations:** Division of Pediatrics, The University of Texas M. D. Anderson Cancer Center, Houston, TX 77030, USA

## Abstract

Ewing's sarcoma accounts for a disproportionately high portion of the overall pediatric mortality rate compared to its rare incidence in the pediatric population. Little progress has been made since the introduction of traditional chemotherapies, and understanding the biology of the tumor is critical for developing new therapies. Ewing's sarcomas rely on a functional vascular supply, which is formed by a combination of angiogenesis and vasculogenesis. Recent insights into the molecular regulation of bone marrow (BM) cell participation in vascular development have identified VEGF, SDF-1*α*, and DLL4 as critical players in the vasculogenesis process. Clinical trials using vascular targeting agents, specifically targeting VEGF or DLL4, are underway.

## 1. Introduction

The current standard of care for Ewing's sarcoma includes a combination of preoperative and adjuvant chemotherapy along with local control. Surgery is the preferred method of local control, but in cases where surgical resection is not possible, radiation therapy is used [1, 2]. First-line chemotherapy includes a combination of five drugs: vincristine, cyclophosphamide, doxorubicin, ifosfamide, and etoposide [3]. This combination therapy achieves a nearly 70% survival rate for patients with nonmetastatic disease [3]. It is presumed that the majority of patients with Ewing's sarcoma have metastatic disease at diagnosis. However, only 25% have detectable metastatic disease using conventional diagnostic methods. The outcome of patients with metastatic disease continues to be poor, with less than 25% expected to be long-term survivors [1, 3].

There is an urgent need for new therapeutic options for patients with Ewing's sarcoma, particularly for those with metastatic disease, as cure rates have remained stagnant for more than 20 years. The most significant progress that has been made is in using dose-intensive chemotherapy which brings multiple toxicities with it [2–5]. A recent study conducted by the Children's Oncology Group demonstrated an increase in event-free 3-year survival for patients with nonmetastatic Ewing's sarcoma by increasing the frequency of chemotherapy. The event-free survival rate was 76% for patients receiving chemotherapy every 2 weeks compared to 65% for patients given standard every-3-week chemotherapy [6]. Understanding the biologic properties of Ewing's sarcoma and what is important in the microenvironment to promote tumor growth will allow the identification of more targeted therapies outside of traditional chemotherapy. 

The development of a functional vascular system is a hallmark of solid tumors [7]. Ewing's sarcomas, like other solid tumors, are reliant on a functional vascular network for the delivery of nutrients and oxygen and for the removal of waste [8]. Therefore, one possible way to inhibit tumor growth and promote tumor regression is by preventing the tumor from developing a vascular supply, essentially starving the tumor of nutrients and oxygen. Defining the molecular mechanisms that direct blood vessel formation in Ewing's sarcoma is key to identifying therapeutic targets that might prevent vascular development. This paper will provide a comprehensive summary of the current understanding of vasculogenesis in Ewing's sarcoma and one of the processes involved in blood vessel formation, as well as a brief review of clinical trials using vascular targeting agents.

## 2. Angiogenesis and Vasculogenesis

In most blood vessels, the lumen, or inner tube through which blood flows, is made up of a single layer of endothelial cells. In arteries and veins, these endothelial cells are surrounded by one or several layers of mural cells. Mural cells include pericytes and vascular smooth muscle cells (vSMCs). Varying amounts of connective tissue, including collagen, elastic fibers, and other proteins and polysaccharides, are interspersed between the cells and surround blood vessels.

At least two processes contribute to blood vessel formation in Ewing's sarcoma: angiogenesis and vasculogenesis. Angiogenesis is the sprouting of preexisting vessels to form new ones. During angiogenesis, endothelial cells and pericytes/vSMC proliferate and migrate, so that new blood vessels would be formed by a locally derived cell population. Alternatively, vasculogenesis is the process by which bone-marrow-(BM-) derived precursor cells are recruited to sites of developing vasculature and organized to form a vessel network *de novo*. Vasculogenesis occurs during embryogenesis and refers to the initial formation of the vasculature. Originally, it was believed that postnatal vascular development occurred only by angiogenesis. However, several studies have highlighted the important role of vasculogenesis in postnatal processes such as the healing of cutaneous wounds, in response to ischemia, and more recently, in tumor growth [9–12]. Angiogenesis and vasculogenesis both contribute to vascular growth in adults, and the level of contribution of each varies based on the tissue and stimulus [13]. In Ewing's sarcoma, the vascular network is formed by a combination of angiogenesis and vasculogenesis. BM-derived cells within Ewing's sarcoma vasculature were first reported by Bolontrade et al., who demonstrated that at least 10% of blood vessels in Ewing's sarcoma xenografts contain one or more BM-derived endothelial cell [14]. This was shown using a BM transplant model that takes advantage of MHC haplotype differences between donor and recipient mice, allowing for the tracking of BM-derived cells within the tumor. Donor BM cells expressed the endothelial marker CD31 and were incorporated into the endothelial layer of blood vessels within the tumor.

Further exploration using various transplant models where BM-derived cells within the tumor were tracked by fluorescent dyes or by transgenically expressed GFP confirmed Bolontrade's report of BM-derived endothelial cells within Ewing's tumor vasculature and expanded this finding to include BM-derived pericytes/vSMCs. While only a small portion of the endothelial cells in Ewing's sarcoma tumor vessels are BM derived, a much larger portion of the pericytes/vSMCs are BM derived. The pericytes/vSMC component of the majority of the blood vessels in Ewing's sarcoma is a mosaic of BM-derived and locally derived cells, indicating a combination of angiogenesis and vasculogenesis [15–17]. TC71 Ewing's sarcoma cells grown subcutaneously in nude mice that had received GFP^+^ BM transplants were used to demonstrate that BM-derived cells are intertwined with locally derived cells in tumor vessels. Analysis of these tumors revealed thick layers of GFP^+^ BM-derived cells surrounding CD31^+^ endothelial cells. Costaining for GFP and the pericyte/vSMC markers, desmin, alpha smooth muscle actin (*α*-SMA), and platelet, derived growth factor receptor beta (PDGFR-*β*), demonstrated that many of the GFP^+^ BM-derived cells also expressed pericyte/vSMC markers, indicating that BM cells differentiate into pericyte/vSMC in Ewing's sarcoma *in vivo* [16]. Importantly, the GFP^+^ BM-derived pericytes/vSMC were adjacent to and intertwined with GFP^−^, locally derived pericytes/vSMC, indicating a contribution from both local pericyte/vSMC proliferation and BM cell differentiation. Recently, BM-derived cells were also shown to be within the vasculature of a xenograft model of Ewing's sarcoma lung metastasis, indicating that vasculogenesis may play a role in vascular formation in metastases as well as primary Ewing's tumors [18].

In addition to endothelial cells and pericytes/vSMC, a small subpopulation of tumor infiltrating BM cells in Ewing's sarcoma are monocytes (CD14^+^) or macrophages (F4/80^+^). However, these cells do not directly participate in the formation of the vessel structure as they are found in the center of the tumor away from the vessels [16]. These nonvascular BM-derived immune cells arise from the Sca1^−^Gr1^+^ mouse BM or CD34^−^ human progenitor cell subpopulations, while Sca1^+^Gr1^+^ mouse BM or CD34^+^ human progenitor cells contribute to the endothelial cell and pericyte/vSMC populations within the tumor [16]. The function of these macrophages in Ewing's sarcoma is unknown at this time. In other types of tumors, macrophages have been demonstrated to play diverse roles including both tumor-promoting and growth-inhibiting effects. A subset of tumor-associated macrophages has been demonstrated to be induced by hypoxia to secrete angiogenic factors that stimulate tumor vascular development [19, 20]. However, the role of macrophages in vascular development in Ewing's sarcoma is not well studied and is beyond the scope of this review.

## 3. Vasculogenesis Is Essential for Ewing's Sarcoma Tumor Growth

The BM-derived cell contribution to Ewing's vessel development is essential for tumor growth. Angiogenesis alone is not sufficient to form a vascular network large enough to support tumor growth. This was demonstrated by the use of an MEKK3 knockout BM transplant model where nude mice (MEKK3 wild type) received MEKK3^−/−^BM transplants [21]. MEKK3 is a mitogen-activated protein kinase kinase kinase that is essential for early vascular formation. BM cells lacking MEKK3 cannot participate in vascular formation. In this model, local endothelial cells and pericytes/vSMC in the recipient mice are MEKK3^+/+^ and can participate in vascular formation while the BM cells, which are MEKK3^−/−^, cannot. Thus, MEKK3^−/−^ BM-transplanted mice have impaired vasculogenesis but normal angiogenesis. The growth of both TC71 and A4573 Ewing's sarcoma tumors was significantly inhibited in MEKK3^−/−^ BM-transplanted mice compared to tumors in control transplanted mice [21]. The inhibition of tumor growth due to the absence of BM cell participation in vascular formation demonstrates the necessity of vasculogenesis for Ewing's sarcoma tumor growth *in vivo*.

## 4. Molecular Controls of Vasculogenesis

### 4.1. BM Cell Recruitment to the Tumor

**Figure 1:**
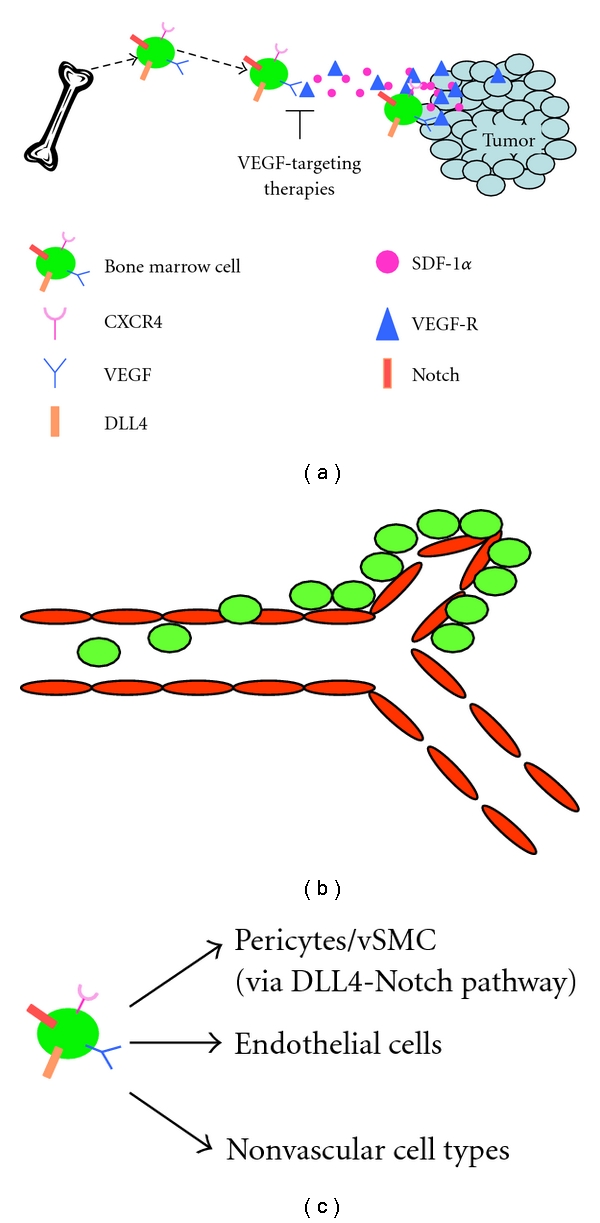
*The steps for BM cell participation in tumor vessel development and expansion. *(a)* BM cell recruitment to the tumor.* One major chemoattractant for BM cell recruitment to the tumor is VEGF_165_. VEGF_165_ is secreted by tumor cells to form a concentration gradient in the circulation, which attracts BM cells. When VEGF_165_ is silenced by siRNA, the number of BM-derived pericytes/vSMCs within tumor vasculature is significantly reduced and tumor growth is inhibited. In the absence of VEGF_165_, SDF-1*α* can recruit BM-derived cells to the tumor and rescue tumor growth. (b)* Adhesion and Extravasation.* When BM cells reach the developing tumor vasculature, they must adhere to the endothelial cell wall and extravasate to the extraluminal side of the vessel. (c)* Differentiation.* Once a BM cell has extravasated to the extraluminal side of a blood vessel within the tumor, it must differentiate from an immature progenitor cell into a mature pericyte/vSMCs, endothelial cell, or nonvascular cell. DLL4-Notch signaling is critical for BM cell differentiation into periyctes/vSMCs in Ewing's sarcoma. BM cells express DLL4 as well as Notch receptors. When DLL4 is inhibited by shRNA or by a DLL4-neutralizing antibody, the number of BM-derived pericytes/vSMCs within the tumor is significantly inhibited and tumor growth is reduced.

BM cell participation in vasculogenesis is a complex, multistep process. BM progenitors must be recruited to the tumor, retained at sites of developing vasculature, and then be directed to differentiate into endothelial cells and pericytes/vSMCs (Figure 1). VEGF_165_ is one of the major chemotactic stimulant for the recruitment of BM-derived cells to Ewing's tumors [22]. VEGF is highly expressed in primary human Ewing's tumors, the serum of Ewing's patients, and Ewing's xenograft tumors [23, 24]. To demonstrate the chemotactic properties of VEGF_165_, shRNA was used to specifically inhibit the expression of VEGF_165_ (but not other VEGF isoforms) in TC71 Ewing's sarcoma cells, creating the stable TC/siVEGF_165_ clone. Prior to the injection of TC/siVEGF_165_ cells, mice received BM transplants of CD34^+^ human cord blood progenitor cells. This allowed for the tracking of the BM cells, as they were human. TC/siVEGF_165_ cells grown subcutaneously in the CD34^+^-transplanted mice formed smaller, slower-growing tumors than parental TC71 or TC/si control cells. Importantly, TC/siVEGF_165_ tumors had decreased microvessel density and reduced numbers of CD34^+^ BM-derived cells [25]. In subsequent studies, selective expression of VEGF_165_, VEGF_189_, or *β*-gal control was induced in TC/siVEGF_165_ tumors using adenoviral vectors. Fluorescently labeled mouse BM cells were subsequently injected into the tumor-bearing mice. Adenoviral VEGF_165_, but not VEGF_189_ or *β*-gal, rescued tumor growth to a level that was similar to wild-type TC71 tumors and caused increased infiltration of BM-derived cells into the tumors. These BM cells participated in tumor vascular formation [22]. Together, these studies demonstrate the essential role of VEGF_165_ in recruiting BM-derived cells to the tumor and in the process of vasculogenesis [22]. 

The important role of VEGF_165_ in BM cell recruitment and tumor growth suggests that VEGF may be a relevant target for the treatment of Ewing's sarcoma. Indeed, subsequent studies using the MHC mismatch BM transplant model demonstrated the efficacy of the VEGFR2 antibody DC101 for preventing BM cell migration into the tumor and inhibiting the growth of Ewing's sarcoma tumors *in vivo *[26]. Tumors from mice treated with DC101 had reduced numbers of BM-derived pericytes/vSMCs, reduced microvessel density, and importantly, significantly-inhibited tumor growth [26].

While VEGF_165_ is chemotactic for BM cells, it also stimulates the local angiogenesis process. The importance of BM cells in the formation and expansion of tumor vessels and the potential of these cells to rescue tumor growth even in the absence of an angiogenesis stimulus was further demonstrated by treating VEGF_165_ inhibited TC71 tumors (TC/siVEGF_165_) with stromal cell-derived factor-1 alpha (SDF-1*α*). SDF-1*α* is a chemoattractant for CXCR4^+^ BM progenitor cells, but has little or no effect on local angiogenesis and does not stimulate tumor cell proliferation. Therefore treating VEGF_165_-inhibited tumors with SDF-1*α* should increase BM cell migration into the tumor area without stimulating local angiogenesis. Treatment of TC/siVEGF_165_ tumors with intratumoral injections of an adenoviral vector carrying the SDF-1*α* gene (Ad-SDF-1) increased tumor expression of SDF-1*α* without increasing VEGF_165_ [27]. In these studies, bilateral TC/siVEGF_165_ cells were implanted into both flanks of nude mice that had received GFP^+^ BM transplants. Tumors were then treated with intratumor injections of Ad-SDF-1 (right side tumors) or Ad-control (left side tumors). Since the tumors were in the same mouse, the host and BM cell pool were identical. Differences in tumor growth or vascular morphology were therefore secondary to the treatment of the tumors and the difference in SDF-1*α* levels. Ad-SDF-1*α* tumors had significantly larger lumen-bearing vessels with increased numbers of GFP^+^ BM-derived pericytes/vSMCs than Ad-control treated tumors. In addition to increased vascularity and BM-derived pericytes/vSMCs, Ad-SDF-1 rescued tumor growth in the absence of VEGF_165_. Ad-SDF-1*α*-treated tumors were larger than their Ad-control counterparts [27]. These data also reinforce the importance of BM-derived pericytes/vSMCs for tumor growth *in vivo* and their ability to circumvent the antitumor activity of agents that solely target VEGF.

### 4.2. BM Cell Differentiation

The process of vasculogenesis requires steps beyond BM cell migration into the tumor. Once BM cells are recruited to the tumor by VEGF_165_, they must differentiate into endothelial cells or pericytes/vSMCs in order to participate in blood vessel formation and help form a functional vessel. As previously stated, the vast majority of vessel-associated BM-derived cells within Ewing's tumors had differentiated into pericytes/vSMCs. Pericytes/vSMCs have several functions relating to vessel structural stability, regulation of blood flow, and vessel maturation [28–30]. Additionally, pericytes/vSMC provide proliferation or quiescence signals to endothelial cells [30, 31]. Without pericytes/vSMC, vessels become leaky and less functional and are more susceptible to regression [31, 32]. Understanding the signals that direct BM cell differentiation into pericytes/vSMC may identify molecular targets that are important for the formation of the BM-derived pericytes/vSMC cell layer that can be targeted to impair tumor vessel functionality which will subsequently inhibit tumor growth.

One pathway found to be important in the differentiation of BM cells into pericytes/vSMC is the Notch signaling pathway. The Notch family is an evolutionarily conserved group of ligands and receptors that regulate diverse biologic processes including cell fate assignment, stem cell maintenance, and boundary formation during development [33]. The mammalian Notch family includes four heterodimeric transmembrane receptors, Notch 1–4, and five transmembrane ligands, Jagged 1, 2 and Delta like ligand 1, 3, 4 (DLL1-4) [33, 34]. Activation of the Notch pathway occurs by direct contact between a membrane bound ligand on one cell and a membrane bound receptor on a neighboring cell [34].

The Notch ligand Delta like ligand 4 (DLL4) is essential for the formation of BM-derived pericytes/vSMC in Ewing's sarcoma *in vivo *[35]. DLL4 is expressed by the BM-derived pericytes/vSMC in Ewing's tumors [35]. This was demonstrated using TC71 or A4573 Ewing's sarcoma xenografts and a GFP BM transplant model. In both A4573 and TC71 tumors, immunohistochemical analysis demonstrated co-localization between DLL4 and GFP, as well as DLL4 and the pericyte/vSMC markers desmin, NG2, and *α*-SMA. Twelve of fourteen human Ewing's sarcoma samples, including metastatic lesions, had similar patterns of DLL4 expression by perivascular cells. DLL4 inhibition by shRNA or by the DLL4 neutralizing antibody YW152F significantly reduced the number of BM-derived pericytes/vSMC within the tumor, reduced vessel functionality (indicated by increased hypoxia), and inhibited tumor growth *in vivo* [35, 36]. The loss of BM-derived pericytes/vSMC after DLL4 inhibition substantiates the critical role of DLL4 in the formation of BM-derived pericytes/vSMCs, and a functional vasculature. DLL4 may therefore be a therapeutic target for the treatment of patients with Ewing's sarcoma, as indicated by the tumor growth inhibition in tumors when DLL4 was blocked.

## 5. Clinical Trials Using Vascular-Targeting Agents

A phase I trial in the Children's Oncology Group (COG) examining bevacizumab, a monoclonal antibody that inhibits VEGF-A signaling, as a single agent was completed. In this study, bevacizumab was administered every two weeks in 28-day courses to children with solid refractory tumors, including 5 patients with Ewing's sarcoma. Two of the 5 had stable disease for greater than 4 months, and one had stable disease for two months. No dose-limiting toxicities occurred [37]. Bevacizumab is already FDA approved for use in patients with a variety of cancer types, including colorectal cancer and glioblastoma multiforme. Concerns over toxicity recently prompted a review of fatal adverse effects (FAEs) related to bevacizumab, in which the authors concluded that while bevacizumab is associated with increased FAEs when combined with taxane-or platinum-based chemotherapies (3.3% v 1.0%), it is not associated with increased FAEs when used in combination with other chemotherapies (0.8% v 0.9%) [38]. 

In addition to VEGF, DLL4 is currently being evaluated as a therapeutic target for the treatment of solid tumors, including Ewing's sarcoma. A phase I trial of the DLL4-neutralizing antibody REGN421 is currently underway (ClincalTrials.gov id: NCT00871559). A trial evaluating REGN421 in pediatric populations is planned.

It is unlikely that antivascular therapies will be effective as single agents against bulk disease. In most preclinical models evaluating VEGF-targeting therapies in Ewing's xenografts, tumor growth was stopped or slowed by treatment, but tumors did not regress and often rebounded after cessation of therapy [24, 39]. One notable exception demonstrated significant regression of SK-NEP-1 primary Ewing's tumors, decreased numbers of lung metastases, and smaller lung metastases in mice treated with VEGF-Trap [40]. A phase I trial using VEGF-Trap, a soluble VEGF decoy receptor, is in progress (Protocol id ADVLO714). However, vascular-targeting therapies will most likely be useful either in combination with chemotherapy or radiation therapy, or as adjuvant therapy following chemotherapy or surgery. While inhibition of DLL4 leads to ablation of the functional vascular network, inhibition of VEGF can, in some cases, lead to a transient stage of vessel normalization. During this stage, vessels are actually more efficient at delivering oxygenated blood. This “vessel normalization” stage may be useful for preparing tumors for radiation therapy, which works most efficiently on well-oxygenated cells, or for combination therapy with chemotherapeutic agents, which would be delivered more efficiently during this phase. It is important to note, however, that long-term anti-VEGF therapy eventually leads to such extensive vessel pruning that even “normalized” vessels are eradicated and tumor growth is inhibited [41]. For an in-depth review of the subject, see the review by De Bock et al. [41]. A phase II trial combining bevacizumab with combination of chemotherapies has recently been discontinued after accruing only 10% of the original target number of participants, making conclusions on the efficacy of bevacizumab and chemotherapy in Ewing's sarcoma difficult to achieve at this time (Children's Oncology Group Trial COG-AEWS0521).

As an alternative to using vascular-targeting agents concurrently with chemotherapy, vasculogenesis inhibitors (such as VEGF or DLL4 inhibitors) may be useful after chemotherapy is completed. After cytotoxic therapy, the number of circulating BM-derived progenitor cells increases [42]. As evidenced by the studies described above using SDF-1*α*, these circulating BM cells may gain access to the tumor and aid in the expansion of residual tumor cells or microscopic metastases by contributing to the formation of a vascular system to feed the metabolic needs of proliferating tumor cells. Therefore, inhibition of VEGF or DLL4, both of which are important for BM cell participation in vasculogenesis, after chemotherapy may prove to be useful in preventing disease relapse or metastasis.

## 6. Conclusion

The contribution of BM-derived cells to the endothelial cell and pericyte/vSMC populations within Ewing's sarcoma vasculature has now been demonstrated using multiple methods to track BM-derived cells within the tumor. Additionally, genetic targeting of vasculogenesis, but not angiogenesis, via MEKK3 knockout has shown that vasculogenesis is essential for the growth of Ewing's sarcoma *in vivo *[21]. Without vasculogenesis, tumor growth is significantly inhibited. Agents targeting VEGF, the major chemotactic stimulus for recruiting BM cells to the tumor, and DLL4, an important molecular signal for the differentiation of BM-derived cells into pericytes/vSMCs, are currently being evaluated for clinical efficacy. Initial studies suggest that SDF-1*α* may also be an important target for the inhibition of vasculogenesis, particularly in tumors that have developed resistance to VEGF-targeted therapy.

While significant progress has been made in understanding the process of vasculogenesis in tumor biology over the past decade, several questions remain. Further understanding of the regulation of vasculogenesis in Ewing's sarcoma will most likely be required for the development of new therapies. For example, the molecular signals controlling BM cell adhesion to tumor vasculature and extravasation to the extraluminal side of blood vessels may also provide therapeutic targets for inhibiting vasculogenesis. Additionally, while DLL4-Notch signaling is important for BM cell differentiation into pericytes/vSMCs, the relative contribution of other pericyte/vSMC differentiation factors, such as transforming growth factor beta (TGF-*β*) and PDGFR-*β*, has not been evaluated in Ewing's sarcoma. Further laboratory studies of the molecular controls of BM cell participation in vasculogenesis as well as clinical studies of specific vascular-targeting agents are the next steps in assessing the potential for using this therapeutic approach in the treatment of patients with Ewing's sarcoma.
